# Validation and optimal cut-off score of the World Health Organization Well-being Index (WHO-5) as a screening tool for depression among patients with schizophrenia

**DOI:** 10.1186/s12888-024-05814-z

**Published:** 2024-05-23

**Authors:** Feten Fekih-Romdhane, Fadila Al Mouzakzak, Ghinwa Abilmona, Oussama Dahdouh, Souheil Hallit

**Affiliations:** 1 Faculty of Medicine of Tunis, Tunis El Manar University, Tunis, Tunisia; 2 The Tunisian Center of Early Intervention in Psychosis, Department of psychiatry “Ibn Omrane”, Razi hospital, Manouba, 2010 Tunisia; 3 Faculty of Science, Lebanese University, Fanar, Lebanon; 4 Research Department, Psychiatric Hospital of the Cross, P.O. Box 60096, Jal Eddib, Lebanon; 5 School of Medicine and Medical Sciences, Holy Spirit University of Kaslik, P.O. Box 446, Jounieh, Lebanon; 6 Department of Psychology, College of Humanities, Effat University, 21478 Jeddah, Saudi Arabia; 7 Applied Science Research Center, Applied Science Private University, Amman, Jordan

**Keywords:** WHO-5, Schizophrenia, Screening, Depression, Psychometric properties, Arabic

## Abstract

**Background:**

The utility of the World Health Organization Wellbeing Index (WHO-5) as rapid screening tool for depression has not yet been researched in the context of schizophrenia. The goals of this study were twofold: (1) to test the psychometric properties of the WHO-5 in a sample of Arabic-speaking patients with schizophrenia from Lebanon, with particular emphasis on validating the WHO-5 as a screening tool for wellbeing and depression in patients with schizophrenia; and (2) to determine the optimal cut-off point to identify schizophrenia patients with depression.

**Methods:**

Chronic, remitted patients with schizophrenia took part in this cross-sectional study between August and October 2023 (*n* = 117; mean age of 57.86 ± 10.88 years and 63.3% males). The Calgary Depression Scale for Schizophrenia (CDSS) was included as index of validity. For the validation of the WHO-5 scale, we performed a confirmatory factor analysis (CFA) using the original structure of the scale. To assess the discriminatory validity of the Arabic version of the WHO-5 as a screening tool for depression, we conducted a Receiver operating characteristic (ROC) curve analysis, taking the WHO-5 reversed score against the dichotomized CDSS score at a cut off value of 6.

**Results:**

The results of CFA supported the originally proposed unidimensional structure of the measure, with good internal consistency reliability (α = 0.80), concurrent validity, and cross-sex measurement invariance. The WHO-5 showed a sensitivity of 0.8 and a specificity of 0.7 in the detection of depression with a cut-off point of 9.5. The validity of the WHO-5 as a screening tool for depression was supported by the excellent discrimination AUC value of 0.838. Based on this WHO-5 cut-off value, 42.6% of the patients were screened as having a depression.

**Conclusion:**

The study contributes to the field by showing that the WHO-5 is a concise and convenient self-report measure for quickly screening and monitoring depressive symptoms in patients with schizophrenia. It is therefore highly recommended to apply this cut-off point for screening and follow-up assessments. The current findings will hopefully encourage clinicians and researchers working in Arab settings, who are often confronted with significant time and resource constraints, to start using the WHO-5 to aid their efforts in mitigating depression in this vulnerable population and fostering research in this under-researched area.

## Introduction

Schizophrenia is one of the leading causes of disability worldwide [1]. It is now largely acknowledged that depression is considered to be a separate entity that is commonly encountered in schizophrenia, with an estimated pooled prevalence of 28.6% [2].

### Depression in schizophrenia

The occurrence of depression in patients with schizophrenia causes substantial family, social, and economic burdens. Depression in schizophrenia negatively affects quality of life [3], and leads to increased suicide risk [4], as well as higher utilization of health services and criminal justice systems [5]. In addition, there is evidence that depression is commonly comorbid with anxiety, and that both conditions are significantly correlated with each other in patients with schizophrenia [6]. Prior research has also demonstrated that depression is a significant predictor of social functioning decline [7] and worse long-term global functional outcomes [8] in people diagnosed with schizophrenia. Despite its high prevalence and significant clinical and socioeconomic impacts, it remains under-detected and inadequately treated [9]. According to a recent meta-analysis [10], the severity of depressive symptoms remains persistent and shows no more than modest improvement in the course of illness regardless of the follow-up length for patients with schizophrenia spectrum disorders. According to the British Association for Psychopharmacology guidelines on the treatment of schizophrenia, the treatment of comorbid depressive symptoms is not receiving the warranted attention given how often they occur [11]. The National Institute for Health and Care Excellence (NICE) guidelines have pointed to the importance of routinely monitoring patients with schizophrenia for a possible coexisting depression [12]. This suggests that strategies for more effective monitoring, evaluation and management of depression in schizophrenia, especially in resource-limited settings, is needed. To this end, accurate measurement instruments which enable assessing depression in this specific population are essential.

### Measurement instruments of depression in patients with schizophrenia

A systematic review of instruments available to evaluate depression occurring in the context of schizophrenia could identify five clinician-rated (i.e., the Montgomery Asberg Depression Rating Scale, the Hamilton Rating Scale for Depression, the Brief Psychiatric Rating Scale—Depression subscale, the Positive and Negative Syndrome Scale—Depression subscale, the Calgary Depression Scale for Schizophrenia [CDSS]) and only one self-report (i.e., the Beck Depression Inventory) measures reliable [13]. Although strong evidence indicates that the CDSS outperforms other depression tools in terms of validity and reliability in patients with schizophrenia [13, 14], it may require a considerable amount of time and interviewers’ training to be completed. For this reason, and despite recommending the use the CDSS as the most reliable and valid for the assessment of depressive symptoms of patients with schizophrenia in both daily clinical practice and in research, Lako et al. [13] called in their meta-analysis for the development of a novel valid self-report measure that can be more expedient for use in clinical practice. A more adequate and efficient alternative that could be considered for repeated assessments in which time and resources are critical factors is the self-report Five-item World Health Organization Well-being Index (WHO-5) [15].

### The psychometric potential of the WHO-5 in patients with schizophrenia

The WHO-5 is a validated global rating measure initially designed to evaluate self-reported well-being in primary health care patients [15]. This shorter version was created from a longer 28-item original version [16] which was employed in a WHO multicentre study conducted in eight European countries [17]. The WHO-5 is composed of five positively phrased items (e.g., “I woke up feeling fresh and rested”) with the aim of measuring positive well-being, and scored on a five-point scale rated from 5 (all of the time) to 0 (none of the time). After its release, the WHO-5 has been translated into over 30 languages and became a widely used instrument in research projects around the globe, with considerable evidence supporting its good psychometric properties and utility [18]. The use of the WHO-5 has considerably increased in mental health settings, as it has been growingly considered a valuable patient-reported outcome assessment in a large array of medical settings and an important research measurement instrument in clinical studies [19]. However, the number of research conducted on psychometric properties of the WHO-5 in patients with schizophrenia spectrum disorders remains very limited, despite the measure having been previously applied in schizophrenia research (e.g [20, 21]). The few psychometric research conducted among this patient population showed that the WHO-5 has good validity, reliability and an invariant unidimensional structure across age, sex, and outpatient/inpatient status [22, 23].

Beyond its usefulness in the measurement and monitoring of well-being, the WHO-5 was found to be one of the main measures which has extensively demonstrated sufficient validity to screen for and early detect depression in different clinical and non-clinical populations across several settings, cultures and countries. Indeed, the WHO-5 showed clinical utility in the screening for depressive symptoms in community adults [24], healthcare workers during the COVID-19 [25], older adults residing in nursing homes [26], as well as in various clinical populations, such as patients with diabetes [27], people diagnosed with Parkinson’s disease [28], and adolescents with major depressive disorder [29]. A systematic review of the literature by Topp et al. [18] encompassing 213 studies revealed that the WHO-5 is sensitive and specific screening instrument for depression, and has a very high applicability across research fields. Surprisingly however, the utility of the WHO-5 as rapid screening tool for depression has not yet been researched in the context of schizophrenia. There is evidence to show that cut-off scores for the WHO-5 when used as a screening tool for depression cannot be generalized to different populations and settings [18]. For this reason, there appears the need to validate and determine the best cut-off score of the WHO-5 that predicts schizophrenia patients at risk for depression. As a brief and simple-to-administer tool in busy clinical services, the WHO-5 can help clinicians prevent an additional burden of depression among patients with schizophrenia by early detecting it, especially in low-resources settings in Arab countries like Lebanon.

### Rationale of the present study

Comorbid depression in schizophrenia is found to be more prevalent in middle-income compared to high-income countries [2]. This can be explained by the fact that risk factors for comorbid depression in schizophrenia, such as trauma, social adversity, and medical conditions [8, 30], occur highly frequently in Low-Middle-Income Countries (such as the Arab Middle East and North Africa region [31–33]). For instance, studies found prevalence rates of depression in patients with schizophrenia of 25.6% in Saudi Arabia [34], 30% in Egypt [35], 36.3% in Qatar [34]. More particularly, Lebanon has been witnessing years of conflicts and continuing social, political and economic unrest. The 2020 Beirut blast and the COVID-19 pandemic further deteriorated the situation in Lebanon and significantly impacted people’s mental health [36]. The multilayered crisis has led to multiple negative consequences in Lebanese clinical populations, including an increase in the rates of depressive symptoms [37]. At the same time, prevention and research efforts regarding depression in schizophrenia are still poorly developed or inappropriate in this part of the world [38]. This considerable early detection and intervention gap may be mainly attributed to a very limited number of mental health professionals and a budget allowed for mental health that is “far below the range to promote mental health services” [39].

Providing evidence for the validity (sensitivity and specificity) and cultural appropriateness of an Arabic-language, user-friendly tool such as the WHO-5 in predicting depression in Arab patients with schizophrenia could aid in the planning and implementation of assessments, prevention and interventions throughout the disease course. Therefore, the goals of this study were twofold: (1) to test the psychometric properties of the WHO-5 (in terms of factor structure, internal consistency, sex invariance, and concurrent validity) in a sample of Arabic-speaking patients with schizophrenia from Lebanon, with particular emphasis on validating the WHO-5 as a screening tool for wellbeing and depression in patients with schizophrenia; and (2) to determine the optimal cut-off point to identify schizophrenia patients with depression. To achieve the second objective, the clinician-rated schizophrenia-specific outcome measure “Calgary Depression Scale for Schizophrenia” (CDSS [40]), is included as index of validity.

## Methods

### Sample and procedure

This cross-sectional study has been conducted during August and October 2023 using convenience sampling. The target sample was set as inpatients of the Psychiatric Hospital of the Cross, Jal Eddib (suburbs of the capital Beirut), Lebanon, with the following inclusion criteria: (1) age of 18 years and over, (2) with a schizophrenia or a schizoaffective disorder diagnosis following the Diagnostic and Statistical Manual of Mental Disorders (DSM-5) criteria; (3) at chronic stage of the disease, defined as with more than 1 year of illness duration [41]; and institutionalized in the above-mentioned long-stay hospital for more than one year (The detailed description of the study population can be found elsewhere [32, 42]); (4) experiencing either partial or total recovery, this choice has been made as personal recovery represents a longitudinal process occurring in stages [43]; and (5) able to give their free and informed consent to participate after study objectives and general instructions were thoroughly explained to them (in case of inability to consent a family member did). This target population was chosen so that potential confounding effects of some factors, such as severity of psychotic symptoms, treatment adherence and substance use, are reduced or eliminated. 

#### Minimal sample size calculation

A sample between 15 and 100 participants was needed for the confirmatory factor analysis based on a previous study that suggested a minimum sample ranging from 3 to 20 times the number of the scale’s variables [44].

### Measures

#### Demographic and clinical characteristics

Data were gathered during a face-to-face interview of around 30–45 min with all participants. The questionnaire consisted of a first section containing demographic and clinical information, including age, sex, education level, marital status, duration of illness, and duration of hospitalization. In addition, four measures were either self- or interviewer-administered to all participants, including the Arabic validated version of the WHO [45]. The other three measures are the following:

#### The Calgary Depression Scale for Schizophrenia (CDSS)

This is a clinician-administered measure containing a total of 9 items with descriptive anchor points [46]. It specifically assesses depression among patients with schizophrenia and related psychoses. Its validity is well-known in strongly correlating with other measurement instruments of depression. The CDSS has also proven to accurately distinguish between depressive symptoms and extrapyramidal side effects and negative symptoms. The Arabic validated version of the CDSS was used [47] (ω = 0.82 / α = 0.81).

#### The 10-item Staden Schizophrenia anxiety rating scale (S-SARS)

This is a clinician-rated measure composed of ten items, five items measure general anxiety and five other items measure specific anxiety [48]. Each item has six narrative anchor points scored on a scale from 0 to 5, and indicating anxiety severity over the past week and is accompanied by guided questions for use during the interview as to inform the ratings. The Arabic validated version of the S-SARS was used [49] (ω = 0.90 / α = 0.89).

#### The Global Assessment of Functioning Scale (GAF)

This measure evaluates social functioning [50]. A number between 0 and 100 is assigned to each patient, summarizing the rater’ s view of the current degree of impairment in terms of educational, occupational, and/or psychosocial function.

### Data Analysis

We performed a CFA on the total sample using the original structure of the scale. The method of estimation used was Maximum Likelihood. To check if the model was adequate, several fit indices were calculated: the normed model chi-square (χ²/df), the Steiger-Lind root mean square error of approximation (RMSEA), the Tucker-Lewis Index (TLI) and the comparative fit index (CFI). Values ≤ 5 for χ²/df, and ≤ 0.08 for RMSEA, and 0.95 for CFI and TLI indicate good fit of the model to the data [51]. Multivariate normality was not verified (Bollen-Stine bootstrap *p* = .002), therefore Bootstrap analysis was conducted. In case of high modification indices between two items, a correlation was added between the residuals of those items. Adding a correlation between those two residuals is done to improve the model fit when the assumption of uncorrelated errors is violated. It suggests that there are shared sources of variance not accounted for by the specified factors in the model. By adding this correlation, the model becomes more flexible and can better capture the underlying relationships in the data, leading to more accurate representation of the relationships between the observed variables and the latent factors being measured.

#### Sex invariance

To examine sex invariance of the WHO-5 scores, we conducted multi-group CFA [52] using the total sample. Measurement invariance was assessed at the configural, metric, and scalar levels [53]. Configural invariance implies that the latent scales variable(s) and the pattern of loadings of the latent variable(s) on indicators are similar across gender (i.e., the unconstrained latent model should fit the data well in both groups). Metric invariance implies that the magnitude of the loadings is similar across gender; this is tested by comparing two nested models consisting of a baseline model and an invariance model. Lastly, scalar invariance implies that both the item loadings and item intercepts are similar across gender and is examined using the same nested-model comparison strategy as with metric invariance [54]. We accepted ΔCFI ≤ 0.010 and ΔRMSEA ≤ 0.015 or ΔSRMR ≤ 0.010 as evidence of invariance [55].

#### Further analysis

We used Cronbach’s α coefficient and McDonald’s ω and Cronbach’s α coefficients to examine reliability since these two coefficients are used when the data show a normal distribution [56], with values greater than 0.70 reflecting adequate composite reliability. Missing values were replaced by the mean of the item. The WHO-5 scores were considered normally distributed according to their skewness and kurtosis values varying between ± 1 [57]. Consequently, the Student t test was used to compare two means. Pearson test was used to correlate those scores with other scores.

#### Screening accuracy for depression

To assess the discriminatory validity of the Arabic version of the WHO-5 as a screening tool for depression, we conducted a Receiver operating characteristic (ROC) curve analysis using the SPSS software v.26, taking the WHO-5 reversed score against the dichotomized CDSS score 6 [46]. “This curve plays a central role in evaluating diagnostic ability of tests to discriminate the true state of subjects, finding the optimal cut off values, and comparing two alternative diagnostic tasks when each task is performed on the same subject” [58]. The sensitivity and specificity values that show the highest area under the curve (AUC) correspond to the cut off value.

## Results

One hundred seventy-seven patients filled the survey, with a mean age of 57.86 ± 10.88 years and 63.3% males. The majority (30.4%) had a primary and a complementary level of education, whereas 88.1% were single. The mean duration of illness was 35.22 ± 37.34 years, whereas that of the duration of hospitalization was 13.25 ± 10.92 years. Other characteristics of the sample can be found in Table 1.

**Table 1:** Sociodemographic and other characteristics of the patients (*n* = 177)

Gender	
Males	112 (63.3%)
Females	65 (36.7%)
Education level	
Primary	51 (30.4%)
Complementary	51 (30.4%)
Secondary	47 (28.0%)
University	19 (11.3%)
Marital status	
Single	156 (88.1%)
Married	5 (2.8%)
Divorced	13 (7.3%)
Widowed	2 (1.1%)
Age (years)	57.86 ± 10.88
Duration of illness (years)	35.22 ± 37.34
Duration of hospitalization (years)	13.25 ± 10.92

The numbers might not added up to the total due to missing values

### Confirmatory Factor Analysis (CFA) of the WHO-5 scale

The unidimensional model was tested via a CFA in the total sample; results indicated that the fit of the scale was excellent: χ^2^/df = 40.49/5 = 8.10, RMSEA = 0.220 (90% CI 0.160, 0.285), SRMR = 0.082, CFI = 0.856, TLI = 0.712 (Table 2). We noticed a high modification index between items 3 and 4 (= 33.21); when adding a correlation between those residuals, fit indices became excellent as follows: χ^2^/df = 2.75/4 = 0.69, RMSEA = 0.001 (90% CI < 0.001, 0.105), SRMR = 0.020, CFI = 1.000, TLI = 1.013. The reliability was excellent as shown via the alpha (= 0.80) and the omega (= 0.80) coefficients.

**Table 2:** Standardised estimates of factor loadings from the confirmatory factor analysis of the five-item World Health Organization Well-being Index (WHO-5)

Item	Loading factor
1.	0.86
2.	0.74
3.	0.56
4.	0.44
5.	0.63

### Sex invariance of the WHO-5 scale

We were able to show partial invariance across gender at the configural, metric, and scalar levels (Table 3). No statistically significant difference between males and females was found in terms of WHO-5 scores (M = 16.78, SD = 7.28 vs. M = 15.24, SD = 6.85, *t*(146) = 1.24, *p* = .218).

**Table 3:** Measurement invariance of the five-item World Health Organization Well-being Index (WHO-5) across sex in the total sample

Model	CFI	RMSEA	SRMR	Model Comparison	ΔCFI	ΔRMSEA	ΔSRMR
Males	1.000	< 0.001	0.016				
Females	0.903	0.245	0.097				
Configural	0.967	0.089	0.016				
Metric	0.905	0.123	0.062	Configural vs. metric	0.062	0.034	0.046
Scalar	0.864	0.127	0.073	Metric vs. scalar	0.041	0.004	0.011

*Note* CFI = Comparative fit index; RMSEA = Steiger-Lind root mean square error of approximation; SRMR = Standardised root mean square residual

### Concurrent validity

Higher anxiety (*r* = − .50; *p* < .001) and depression (*r* = − .66; *p* < .001) were significantly associated with lower WHO-5 scores, whereas higher levels of functioning (*r* = .55; *p* < .001) were significantly associated with greater WHO-5 scores.

### ROC analysis of the WHO-5 tested against the CDSS

**Fig. 1:**
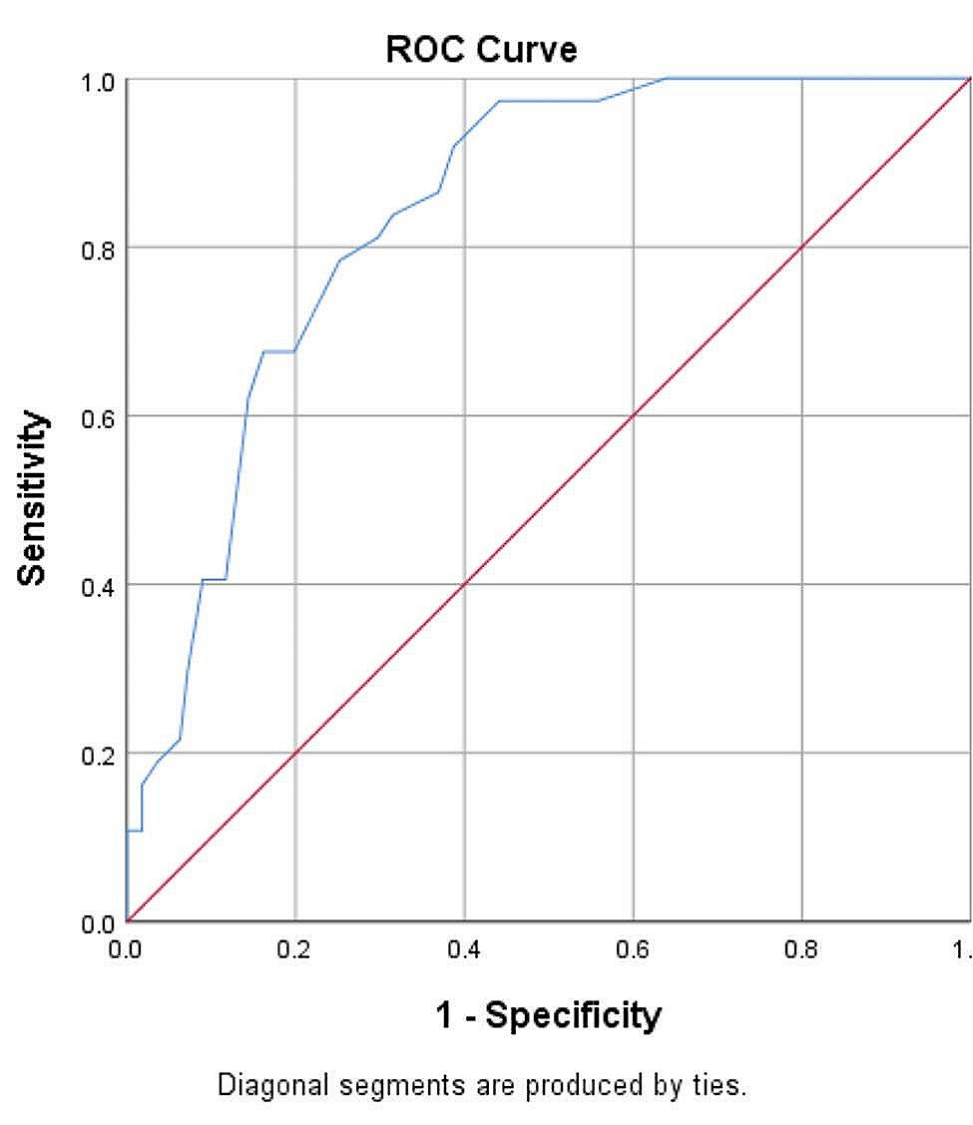
ROC curve for the prediction of WHO-5 scores against CDSS

The ROC curve illustrating depression prediction using the WHO-5 scale is depicted in Fig. 1. It exhibited a substantial area under the curve of 0.838 [95% CI 0.773; 0.903]. Notably, one significant cut-off point was discerned at 9.5, which yielded a sensitivity of 81.1% and a specificity of 70.3%. In selecting this cut-off, higher sensitivity was favoured over specificity. The reason for this choice is that the objective of the WHO-5 as a screening tool in this context is to correctly capture most depressed patients with schizophrenia. As such, a sufficiently high sensitivity is likely to be of significance in screening for depression in this population. Using a WHO-5 cut-off score of 9.5, 42.6% of the patients were screened as having a depression.

## Discussion

The WHO-5 is one of the main measures which has extensively demonstrated sufficient validity to screen for and early detect depression in different clinical and non-clinical populations across several settings, cultures and countries around the globe. Our study offers two new insights. First, it examines psychometric properties of the WHO-5 for the first time in schizophrenia patients from a non-Western developing country and an Arab culture. Second, this is the first research to investigate the potential of the WHO-5 as a screening tool for depression in this clinical population. Our findings demonstrated that the WHO-5 measure in its Arabic version meets the required validity and reliability criteria. In addition, the WHO-5 showed a sensitivity of 0.81 and a specificity of 0.70 in the detection of depression with a cut-off point of 9.5. The validity of the WHO-5 as a screening tool for depression was supported by the excellent discrimination AUC value of 0.838.

### Psychometric properties of the WHO-5 in patients with schizophrenia

First, the goodness of fit for the one-factor model of the Arabic WHO-5 was examined using CFA. The results of these analyses were in agreement with evidence of unidimensionality of the WHO-5 obtained by several validation studies carried-out in various clinical populations, including patients with epilepsy [59], diabetes [27], patients in primary care [60], as well as those in specialised community mental health settings [61]. A recently published validation study among Danish patients with schizophrenia spectrum disorders [22] provided support to the originally proposed unidimensional structure of the measure and good internal consistency reliability (Cronbach’s alpha was 0.80 in our Arabic-speaking Lebanese sample versus 0.826 in the Danish sample). Consistently, previous studies performed among individuals in mental health settings indicated good internal consistency, with Cronbach’s alpha coefficients ranging from 0.83 [62] to 0.92 [63]. Second, and consistent with the same study, measurement invariance of the WHO-5 was established between male and female patients, implying that no differential item functioning exists for sex [22]. Third, the concurrent validity findings revealed that WHO-5 scores were positively correlated with levels of functioning, and inversely correlated with anxiety and depression symptoms’ severity, thereby confirming the findings of earlier research studies [64–66]. Altogether, our psychometric findings were in line with the limited existing literature. A study performed among a sample of Spanish outpatients drawn from specialised community mental health centres (among them, 11.5% were diagnosed with schizophrenia) found that the WHO-5 was feasible to administer, yielded a good fit in a one-factor solution, and showed an excellent internal consistency (Cronbach’s α = 0.923) [61]. Another study involving 84 Chinese individuals diagnosed with schizophrenia, schizotypal and delusional disorders found that the Hong Kong Cantonese Version of the WHO-5 exhibited a single-factor structure and satisfactory internal consistency (Cronbach’s alpha coefficient of 0.86) [23]. Acceptable psychometric parameters of the Farsi WHO-5 were also found among Iranian psychiatric outpatients, with a one-factor structure identified, a Cronbach’s α value of 0.91 and negative correlations with depression measures [67].

### Performance of the WHO-5 as a screening test for depression in patients with schizophrenia

A score of 6 on the CDSS, which is adopted as a valid cut-off indicating levels of depression at which treatment is needed (major depression) [46], corresponded to a WHO-5 score of 9.5. The area under the curve (AUC) values were greater than 0.80, indicating that the WHO-5 is successful as a screening tool to discriminate between patients who are at high risk and low risk for depression [68]. At this cut-off point, the sensitivity and specificity values were 0.81 and 0.70, respectively. Achieving sufficiently high sensitivity for a screening tool such as the WHO-5, so as to be able to correctly identify as many depressed patients as possible as screening positive, is a crucial target and a main challenge of depression detection. However, reaching high specificity, which refers to identifying as few “false positives” as possible (i.e., non-depressed patients who screen positive on the WHO-5) has less importance. This is because the rates of false positives can easily be controlled by a two-step approach screening, with an initial step consisting of an administration of the self-report WHO-5, followed by a structured diagnostic interview administered by trained clinicians. As this is the first study to investigate the optimal cut-off point of the WHO-5 to screen depression among patients with schizophrenia, comparison with previous findings in the same population is not possible. However, similar results were observed in other populations, such as adolescents, in whom cut-off point of nine was recommended, and yielded sensitivity and specificity values of 0.74-0.79 and 0.71-0.89, respectively [69, 70].Using a WHO-5 cut-off score of 9.5, 42.6% of the patients were screened as having a depression. This finding is consistent with that of a meta-analysis of 53 observational studies which highlighted high prevalence estimates of comorbid depression in schizophrenia ranging from 4.6 to 65.1%, with rates being greater in Middle-income than High-income countries (30.2% versus 27.1%, respectively) [2]. Depression is present in all phases of schizophrenia [71], and may have heavy detrimental effects on the course and prognosis of the disease (including more severe psychotic symptoms, longer disease duration [2], and high suicidality risk [4]). However, depression can also be effectively prevented and managed when timely detected [72]. This underscores the high relevance of using the WHO-5 as a brief, reliable, and valid scale, which is suitable for use in routine clinical practice for rapid screening of depression in people with schizophrenia.

### Study limitations

To the best of our knowledge, the present study is among the very few to examine the psychometric properties of the WHO-5 in patients with schizophrenia, and the first to suggest a cut-off value of this tool in this population. Another important strength of this study is that a trained interviewer conducted structured interviews using the CDSS, which is considered a gold standard [73], to determine each patient’s depression level after administration of the self-report WHO-5. In addition, by involving an under-researched population from the Middle-East and the Arab world, our study makes a valuable contribution to the literature. The study has also limitations that need to be recognized. Patients were gathered from a single centre and country by the convenience sampling method; therefore, our findings lack generalisability. To address this limitation, further research should be performed in patients recruited by random sampling and from different countries/settings. Besides, the study sample was composed of a majority of males (63.3%). Future research needs to use a sex-proportionate sample to confirm our findings. In addition, only chronic inpatients residing a long-stay mental hospital were involved, and cannot be representative of the overall schizophrenia population. Future studies need to include outpatients, and those in the early stages of the disease. Another limitation lies to the adoption of a cross-sectional design. As follow-up data was not collected in this study, the test-retest reliability was not assessed. Researchers need to consider collecting data at multiple time points to remedy this limitation.

### Current perspectives and future implications

The present findings provided additional evidence of good psychometric qualities of the WHO-5 in patients with schizophrenia, including good structural validity (unidimensionality), adequate internal consistency, and appropriate concurrent validity. Analyses also demonstrated that measurement equivalence across sexes was achieved, indicating that the WHO-5 measures the same underlying construct in male and female patients with schizophrenia, and that inferences of sex differences in WHO-5 scores are accurate. Overall, findings support the suitability and usefulness of the Arabic version of the WHO-5 in patients with schizophrenia for both clinical and research practices. In addition, the performance of the Arabic-language WHO-5 as a screening tool for depressive symptoms in patients with schizophrenia was tested and supported. It is therefore highly recommended to apply this cut-off point for screening and follow-up assessments. The current findings will hopefully encourage clinicians and researchers working in Arab settings, who are often confronted with significant time and resource constraints, to start using the WHO-5 to aid their efforts in mitigating depression in this vulnerable population and fostering research in this under-researched area. In particular, the WHO-5 can be an appropriate screening and monitoring tool for schizophrenia patients with an elevated vulnerability to depression, therefore enhancing the quality of care provided to this population. Indeed, detecting and monitoring patients’ depression can inform treatment decisions, and subsequently have major positive impact on their clinical outcomes [74]. The brevity of the WHO-5, its ease of use, and validity with the clinician-administered CDSS imply that the scale could be a valuable measure for recognizing comorbid depression in patients with schizophrenia using a cut-point of 9.5.

As for future research perspectives, future studies with larger and more representative samples of patients with schizophrenia in different stages of the disease and originating from different Arab countries are still warranted to confirm our conclusions about the good psychometric qualities of the WHO-5 as a screening tool for depression, and enable its widespread use among Arabic-speaking schizophrenia patients in different parts of the world. Such research might help advance knowledge regarding the prevalence, epidemiology, and cause of depression in patients with schizophrenia, and gain cross-cultural insights into factors that influence the expression of depressive symptoms, their diagnosis and treatment among Arabic-speaking schizophrenia patients.

## Conclusion

The study contributes to the field by offering a brief self-report scale for depression screening and monitoring, the Arabic WHO-5, which is easily accessible, quick-to-administer, simple to understand and practical for use in routine clinical and research practices. As expected, findings suggest that the WHO-5 is an applicable and accurate tool for screening of depression among patients with schizophrenia. Analyses showed that a cut-off score of 9.5 on the WHO-5 can be considered to identify schizophrenia patients with depression. is the study’s results are expected to raise awareness about the necessity to consider depression as a key predictor of outcome and an important therapeutic target in patients with schizophrenia in Arab settings. Henceforth, clinicians and researchers working and dealing with Arabic-speaking schizophrenia patients in different clinical settings worldwide could feel more secure making informed decisions based on this tool.

## Data Availability

All data generated or analyzed during this study are not publicly available. The dataset supporting the conclusions is available upon request to the corresponding author.
